# Genomic neighborhoods for *Arabidopsis *retrotransposons: a role for targeted integration in the distribution of the Metaviridae

**DOI:** 10.1186/gb-2004-5-10-r78

**Published:** 2004-09-29

**Authors:** Brooke D Peterson-Burch, Dan Nettleton, Daniel F Voytas

**Affiliations:** 1 National Animal Disease Center, 2300 N Dayton Ave, Ames, IA 50010, USA; 2 Department of Statistics, 124 Snedecor Hall, Iowa State University, Ames, IA 50011, USA; 3 Department of Genetics, Development and Cell Biology, 1035A Roy J. Carver Co-Lab, Iowa State University, Ames, IA 50011, USA

## Abstract

**Background:**

Retrotransposons are an abundant component of eukaryotic genomes. The high quality of the *Arabidopsis thaliana *genome sequence makes it possible to comprehensively characterize retroelement populations and explore factors that contribute to their genomic distribution.

**Results:**

We identified the full complement of *A. thaliana *long terminal repeat (LTR) retroelements using RetroMap, a software tool that iteratively searches genome sequences for reverse transcriptases and then defines retroelement insertions. Relative ages of full-length elements were estimated by assessing sequence divergence between LTRs: the Pseudoviridae were significantly younger than the Metaviridae. All retroelement insertions were mapped onto the genome sequence and their distribution was distinctly non-uniform. Although both Pseudoviridae and Metaviridae tend to cluster within pericentromeric heterochromatin, this association is significantly more pronounced for all three Metaviridae sublineages (*Metavirus*, *Tat *and *Athila*). Among these, *Tat *and *Athila *are strictly associated with pericentromeric heterochromatin.

**Conclusions:**

The non-uniform genomic distribution of the Pseudoviridae and the Metaviridae can be explained by a variety of factors including target-site bias, selection against integration into euchromatin and pericentromeric accumulation of elements as a result of suppression of recombination. However, comparisons based on the age of elements and their chromosomal location indicate that integration-site specificity is likely to be the primary factor determining distribution of the *Athila *and *Tat *sublineages of the Metaviridae. We predict that, like retroelements in yeast, the *Athila *and *Tat *elements target integration to pericentromeric regions by recognizing a specific feature of pericentromeric heterochromatin.

## Abstract 2

The full complement of *Arabidopsis* LTR retroelements was identified and relative ages of full-length elements estimated showing that Pseudoviridae are much younger than Metaviridae. The distribution of retroelement insertions across the genome was shown to be non-uniform.

## Background

Endogenous retroviruses and long terminal repeat (LTR) retrotransposons (collectively called retroelements) generally comprise a significant portion of higher eukaryotic genomes. Dismissed as parasitic or 'junk' DNA, these sequences have traditionally received less attention than sequences contributing to the functional capacity of the organism. This perspective has changed with the completion of several eukaryotic genome sequences. The contributions of retroelements to genome content range from 3% in baker's yeast to 80% in maize [1,2]. Retroelement abundance has resulted in increased appreciation of the important evolutionary role they play in shaping genomes, fueling processes such as mutation, recombination, sequence duplication and genome expansion [3].

The impact of retroelements on their hosts is not without constraint: the host imposes an environmental landscape (the genome) within which retroelements must develop strategies to persist. Retroelement cDNA insertion directly impacts on the host's genetic material, making this step a likely target for regulatory control. Transposable elements (TEs) in some systems utilize mechanisms that direct integration to specific chromosomal sites or safe havens [4,5]. For example, the LTR retrotransposons of yeast are associated with domains of heterochromatin or sites bound by particular transcriptional complexes such as RNA polymerase III [6-9]. These regions are typically gene poor and may enable yeast retrotransposons to replicate without causing their host undue damage [10]. Non-uniform chromosomal distributions are observed in other organisms as well. For example, many retroelements of *Arabidopsis thaliana *and *Drosophila melanogaster *are clustered in pericentromeric heterochromatin [11,12]. However, beyond the yeast model, it is not known whether retroelements generally seek safe havens for integration.

The genome of *A. thaliana *is ideal for exploring processes that influence the chromosomal distribution of retroelements. *A. thaliana *retroelement diversity has been analyzed previously, preparing the way for this study [13-15]. In contrast to the genomes of *Saccharomyces cerevisiae*, *Schizosaccharomyces pombe *and *Caenorhabditis elegans*, which have relatively few retroelements, *A. thaliana *has a diverse mobile element population whose physical distribution can be described in detail. Another benefit of *A. thaliana *stems from the fact that in contrast to most other 'completely sequenced' eukaryotic genomes, the *A. thaliana *genome sequence better represents chromosomal DNA of all types, including sequences within heterochromatin [11]. Here we undertake a comprehensive characterization of the LTR retroelements in the well characterized genome of *A. thaliana *to better understand the factors contributing to their genomic distribution.

## Results

### Dataset

**Figure 1:**
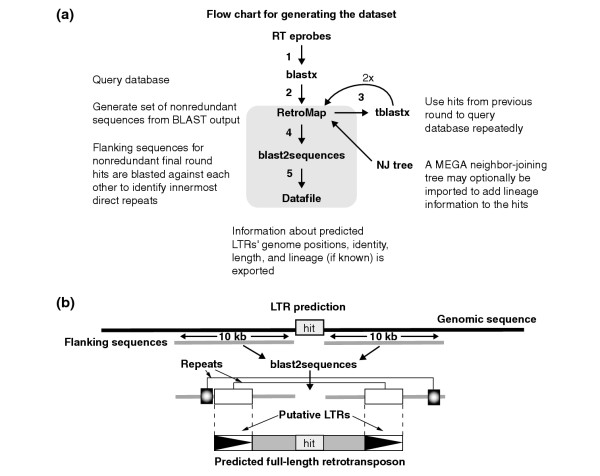
Assembling the retroelement dataset. **(a) **Flow chart for the generation of the dataset. The shaded region denotes steps coordinated by the RetroMap software. (Eprobe refers to a BLAST query sequence) **(b) **LTR prediction. The innermost direct repeats identified in sequences flanking the original BLAST hit are assigned as LTRs. The repeats delimit the boundaries of the full-length LTR retrotransposons.

All reverse transcriptases in the *A. thaliana *genome were identified by iterated BLAST searches (Figure 1). The query sequences were representative reverse transcriptases from the Metaviridae, Pseudoviridae and non-LTR retrotransposons (Table 1). LTRs (if present) were assigned to each reverse transcriptase using the software package RetroMap (Figure 1, see also Materials and methods). Although the coding sequences of many elements with flanking LTRs were degenerate, they are referred to as full-length or complete elements (FLE) to indicate that two LTRs or LTR fragments could be identified. 5' LTRs from FLEs and published *A. thaliana *elements were used to identify solo LTRs in the genome by BLAST searches. The final data set consisted of three insertion subtypes: 376 FLEs, 535 reverse transcriptase (RT)-only hits, and 3,268 solo LTRs (Table 2). These sequences comprise 3,951,101 bases or 3.36% of the total 117,429,178 bases in The Institute of Genomic Research (TIGR) 7 January 2002 version of the genome. Overall, chromosomal retroelement content ranged from 2.64% (chromosome 1) to 4.31% (chromosome 3). Chromosome 4 contained the fewest FLEs (53) and solo LTRs (449), whereas chromosome 3 had the most (92 FLEs and 1,053 solo LTRs).

**Figure 2:**
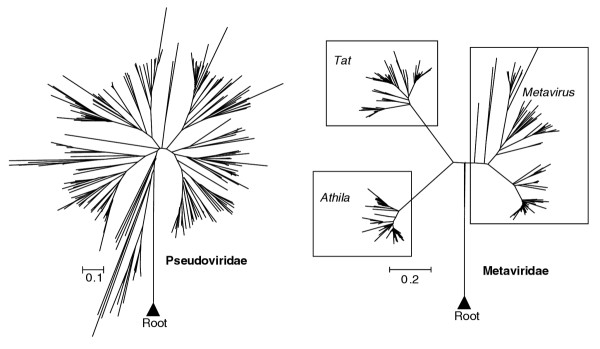
*Arabidopsis thaliana *Metaviridae and Pseudoviridae reverse transcriptase diversity. Phylogenetic trees used in this figure are adapted from [14,18]. Each tree is based on ClustalX [56] alignments of reverse transcriptase domains for elements in a given family. Neighbor-joining trees (10,000 bootstrap repetitions) were generated using MEGA2 [57]. The non-LTR retrotransposon Ta11 served as the root for both trees. The three Metaviridae sublineages are boxed.

Element subtypes (FLE, RT-only and solo LTRs) were sorted into taxonomic groupings using the formal taxonomic nomenclature assigned to retrotransposons [16,17]. Our analysis identified numerous insertions for both the Pseudoviridae (211 FLE/82 RT-only/483 solo LTRs) and Metaviridae (168 FLE/142 RT-only/2,803 solo LTRs). The non-LTR retrotransposons lack flanking direct repeats, and therefore only reverse transcriptase information is provided in this study; 311 non-LTR retrotransposon reverse transcriptases were identified. Unlike the Pseudoviridae, *A. thaliana *Metaviridae elements can easily be divided into sublineages, which are referred to as the *Tat*, *Athila *and *Metavirus *elements [14,18] (Figure 2). Our method identified 42 *Tat *FLEs, 38 *Athila *FLEs and numerous divergent *Metavirus *elements (82 FLE). No evidence was found for *BEL *or *DIRS *retroelements.

The Metaviridae make up 2.34% of the *A. thaliana *genome, whereas the Pseudoviridae represent only 1.25% of the total genomic DNA. This difference is accounted for largely by the longer average size of Metaviridae FLEs (8,952 nucleotides) and solo LTRs (447 nucleotides) when contrasted with the Pseudoviridae FLEs (5,336 nucleotides) and solo LTRs (187 nucleotides) (data not shown). Among the subgroups of the Metaviridae, the average length of *Metaviruses *is closer to that of the Pseudoviridae than to the mean lengths of the *Athila *and *Tat *lineages. The Pseudoviridae are also more uniformly sized than the Metaviridae. A second factor contributing to the abundance of Metaviridae is that they have approximately six times more solo LTRs than the Pseudoviridae, even though numbers of complete elements are similar between families (Table 2). The ratios of solo LTRs to FLEs also clearly differ between the Metaviridae (16.7:1) and Pseudoviridae (2.3:1).

### Chromosomal distribution

**Figure 3:**
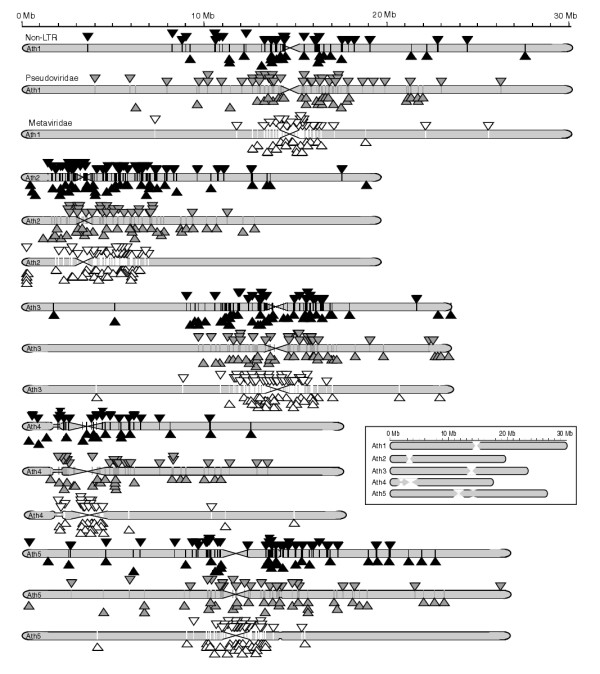
Physical distribution of full-length *A. thaliana *retroelements. The five *A. thaliana *chromosomes are designated as Ath1-5. Triangles indicate the location of a particular retroelement on the chromosome. Non-LTR retrotransposons are in black, Pseudoviridae in gray, and Metaviridae in white. Vertical bars on the chromosome show the precise location of the retroelement. Regions of heterochromatin are represented as follows: telomeres and NORs (on Ath2 and Ath4) by rounded chromosome ends; centromeres by hourglass shapes; heterochromatic knobs (on Ath4 and Ath5) by narrowed stretches on chromosome bars. The relatively short chromosome 5 knob is barely visible to the right of the centromere. The inset more clearly depicts heterochromatic regions that are obscured by element insertions. Chromosomes are drawn to scale.

**Figure 4:**
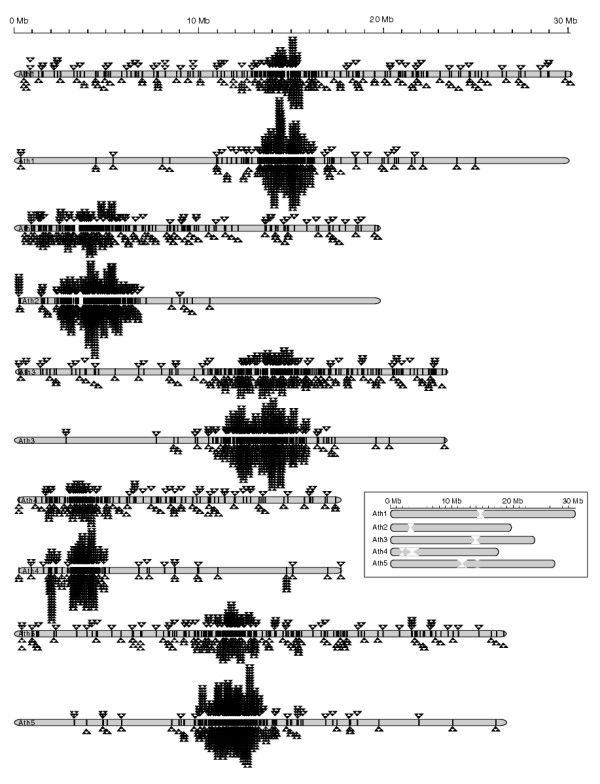
Chromosomal distribution of LTRs for the Metaviridae and Pseudoviridae families in *A. thaliana*. Chromosomes are displayed as in Figure 3. In addition, solo LTRs are drawn as open triangles. The upper chromosome depicts the distribution of Pseudoviridae, the lower the distribution of Metaviridae. In contrast to Figure 3, shading is not used to distinguish between the families.

The distribution of retroelements was examined on a genome-wide basis. Upon mapping the retroelement families onto the *A. thaliana *chromosomes, the previously noted pericentromeric clustering of TEs was immediately evident (Figure 3) [11]. The Metaviridae appeared to cluster in the pericentromeric regions more tightly than the Pseudoviridae and non-LTR retrotransposons. Distributions of these latter two groups appeared similar, as did the distribution of solo LTRs relative to full-length elements (Figure 4).

We assessed statistical support for the apparent clustering of elements by comparing the observed distribution of each lineage to a random uniform distribution model (Table 3). This model assumes that any location in the genome is expected to have a uniform probability of element insertion. This model was rejected by Kendall-Sherman tests of uniformity for every lineage and chromosome combination. All *p*-values were less than 0.05 and most were less than 0.0001.

We next looked at distribution patterns between element families to determine whether they are similar. On the basis of the retroelement distribution maps (Figure 3), we hypothesized that this would not be the case for the Metaviridae because they appeared to be associated with centromeres to a greater degree than the other families. Each family's chromosomal distribution, inclusive of all subtypes (for example, FLE, RT-only and solo LTR), was tested for similarity to the distribution of the other families using a permutation test. With the exception of chromosome 3, the distribution of non-LTR retrotransposons was not significantly different from that of the Pseudoviridae. Comparisons of Metaviridae elements with Psedoviridae and/or non-LTR elements differed significantly (*p *< 0.05) for all combinations.

To assess whether the Metaviridae sublineages contributed equally to the observed distribution bias, we tested a model wherein the three sublineages (*Athila*, *Tat *and *Metavirus*) were expected to have similar distributions. This appears to be true, as significant differences were not detected on any chromosome for these sublineages. We then checked whether the FLEs, RT-only hits or solo LTRs displayed different distributions from one another within their respective families. No consistently significant trends were observed for the Pseudoviridae or the Metaviridae. Oddly, the Metaviridae solo LTR distribution displayed significant differences from the FLEs and RT-only hits for chromosome 3.

A feature of pericentromeric regions in *A. thaliana *is that they are heterochromatic, a state required for targeted integration by the yeast Ty5 retroelement [19]. Because of the observed pericentromeric clustering of retrotransposons in *A. thaliana*, we assessed a simple model that assumes that all elements transpose to heterochromatin (Table 4). There are several genomic regions that are typically considered heterochromatic in *A. thaliana *- centromeres, knobs (on chromosomes 4 and 5), telomeres and rDNA [20-22]. We looked for differences between lineages with respect to whether retroelements were within a heterochromatic region, or, if outside, whether differences existed in distances to the nearest heterochromatic domain. All lineage combinations showed highly significant differences in heterochromatic distributions. In the Metaviridae, the *Metavirus *elements are less tightly associated with heterochromatin than are *Tat *and *Athila*, which did not differ significantly from each other. Element subtypes also differed in their distribution with respect to heterochromatin. The major source of differences was the distribution of solo LTRs in the Metaviridae.

### Age of insertions

**Figure 5:**
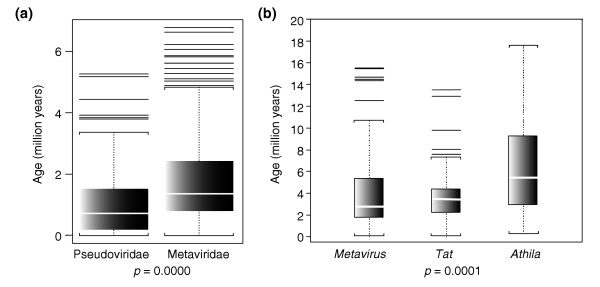
Relative ages of *A. thaliana *LTR retroelement lineages. **(a) **Box-plot showing the age distribution of Pseudoviridae full-length elements contrasted with those of the Metaviridae. The position of the median is shown as a gray bar in the box that delimits the boundaries of the lower and upper quartiles. Data points more than 1.5 times the inter-quartile range above the upper quartile or below the lower quartile are indicated by individual horizontal lines. Ages were calculated as described in Materials and methods. **(b) **Relative-age box-plots of Metaviridae sublineages. Permutation test *p*-values for the significance of the displayed age distributions are shown below each box-plot.

LTR retroelements have a built-in clock that can be used to estimate the age of given insertions. At the time an element inserts into the genome, the LTRs are typically 100% identical. As time passes, mutations occur within the LTRs at a rate approximating the host's mutation rate. LTR divergence, therefore, can be used to estimate relative ages between elements, assuming that all elements share the same probability of incurring a mutation. Although it is possible to estimate ages for non-LTR retrotransposons by generating a putative ancestral consensus sequence and calculating divergence from the consensus, this method is not directly equivalent to estimating ages by LTR comparisons. Therefore, age comparisons were performed only for the LTR retroelement families. Note that the ages depicted in Figure 5 are relative, and we do not claim that a particular element is a specific age in this study. Rather, we focus on whether elements are significantly older or younger than each other.

Statistically significant age differences were observed among the Pseudoviridae and three Metaviridae sublineages (*F *= 14.4, df = 3 and 368, *p *< 0.0001) (Table 5, Figure 5). Overall, the Pseudoviridae are younger than the Metaviridae (*t *= 5.72, df = 368, *p *< 0.0001). When the Metaviridae sublineages are considered, it is apparent that the *Athila *elements are responsible for much of the increased age of this family. The difference between *Athila *and the other two sublineages is significant, with *p *= 0.0003 being the highest value for sublineage comparisons. Elements within heterochromatic regions were significantly older than those found outside (*F *= 17.19, df = 1 and 368, *p *< 0.0001). There was suggestive evidence that the mean element ages varied among chromosomes (*F *= 2.73, df = 4 and 368, *p *= 0.0289). However, all pairwise comparisons between chromosomes failed to yield significant results at the 0.05 level using the Tukey-Kramer adjustment (data not shown).

## Discussion

Completed genome sequences enable comprehensive analyses of retroelement diversity and the exploration of the impact of retroelements on genome organization. Although most large-scale sequencing projects use the shotgun sequencing method, this method makes it particularly difficult to assemble repetitive sequences and to correctly position sequence repeats on the genome scaffold. Consequently, regions of repetitive DNA such as nucleolar-organizing regions (NORs), telomeres and centromeres tend to be skipped, or are sometimes represented by consensus or sampled sequences. The difficulty of cloning repetitive sequences and the drawbacks noted above result in the under- or misrepresentation of the repetitive content of most genomes. Because retroelements frequently comprise a large proportion of the repetitive DNA, 'completed' genome sequences are typically not ideal for studies of retroelement diversity and distribution on a genomic scale. In contrast to these cases, the *A. thaliana *genome is reliably sequenced well into heterochromatic regions and work continues to further define these domains [11,23].

Another factor frustrating comprehensive analyses of eukaryotic mobile genetic elements is the inherent difficulty in annotating these sequences. Many mobile element insertions are structurally degenerate, rearranged through recombination or organized in complex arrays. Software tools and databases such as Reputer [24] and Repbase update [25] have been developed to identify and classify repeat sequences, and these tools have proved helpful in several genome-wide surveys of mobile elements. RECON [26] and LTR_STRUC [27] are software tools that go one step further and consider structural features of mobile elements that can assist in genome annotation. We developed an additional software tool, called RetroMap, to assist in characterizing the LTR retroelement content of genomes. RetroMap delimits LTR retroelement insertions by iterated identification of reverse transcriptases followed by a search for flanking LTRs. The software goes beyond existing platforms and carries out a number of analytic functions, including age assignment, solo LTR identification and visualization of the chromosomal locations of various groups of identified elements on a whole-genome scale.

Data generated by RetroMap are subject to a few caveats. First, because element searches use reverse transcriptase sequences as queries, elements lacking reverse transcriptase motifs (for whatever reason) will not be identified. Second, when RetroMap encounters nested elements, tandem elements, and other complex arrangements, it does not attempt to delimit the element. Rather, the user is notified that a complex arrangement was encountered and the original reverse transcriptase match and any LTR(s) found are logged as separate entities.

For the most part, RetroMap was quite effective in identifying LTR retrotransposon insertions. Our results closely agree with the findings of a parallel study conducted by Pereira [28]. For the Pseudoviridae and two of the three Metaviridae lineages (*Tat *and *Metavirus*), we identified 210 and 128 full-length elements, respectively, whereas Pereira recovered 215 and 130 insertions for these respective element groups. The two studies, however, differed significantly in the number of *Athila *elements identified. We found 38 insertions, whereas Pereira recovered 219. To reconcile these differences, we independently estimated *Athila *copy numbers by conducting iterative BLAST searches with a variety of *Athila *query sequences (data not shown). BLAST hits recovered with each query were then mapped onto the genome sequence. As a result of this analysis, we concluded that RetroMap missed many *Athila *insertions, either because they are highly degenerate or part of complex arrangements. In contrast to Pereira's approach, RetroMap requires that a reverse transcriptase reside between LTRs, and in many cases reverse transcriptases were absent or not detectable in *Athila *insertions. This can be resolved in future implementations of RetroMap that enable multiple query sequences to be tested. The *Athila *elements are large, and our underestimate of the number of *Athila *elements resulted in a corresponding underestimate of the total amount of retrotransposon DNA in the *A. thaliana *genome. We calculated 3.36% for this value, whereas Pereira calculated 5.60%. Pereira's estimate is likely to be the more accurate of the two.

With the exception of the *Athila *elements, the observed frequency of insertions in complex arrangements was rare. For example, the Pseudoviridae had only eight nested and five unassignable elements. The small observed number of complex element arrangements in *A. thaliana *contrasts sharply with observations in grass genomes, where retroelements are usually found in complex nested arrays [29,30]. This may reflect a difference between species in factors contributing to chromosomal distribution of retroelements, or it may simply be a consequence of the difference in abundance of retroelements between *A. thaliana *(5.60% of the genome) and grasses (up to 80% of some genomes) [1,28].

### Genomic distribution of *A. thaliana *retroelements

Our data on the genomic distribution of retroelements can be considered in the light of theoretical work predicting the distribution of TE populations within genomes. These studies largely focus on the effects of selection and recombination on element insertions [31,32]. Particularly relevant is the recent study by Wright *et al. *[33], which considers the effects of recombination on the genomic distribution of major groups of mobile elements in *A. thaliana *(DNA transposons and retroelements). Our analysis extends this work by considering the genomic distribution of specific retroelement lineages. We investigate a model wherein selection and recombination affect element lineages uniformly, and hypothesize that observed deviations in the genomic distribution of specific element lineages reflect unique aspects of their evolutionary history or survival strategies such as targeted integration.

### Ectopic exchange model

The ectopic exchange model assumes that inter-element recombination restricts growth of element populations [31]. Elements should be most numerous in regions of reduced recombination such as the centromeres, because of less frequent loss by homologous recombination. A corollary is that element abundance at a genomic location should inversely reflect the recombination rate for that region in the genome. Previous work suggests that this model is not the primary determinant of element abundance in *A. thaliana*. Wright *et al. *[33] examined recombination rate relative to element abundance in detail and found that the abundance of most *A. thaliana *TE families actually had a small but positive correlation with recombination rate, as was also observed in *C. elegans *[34]. Devos *et al. *[35] found ectopic recombination to be very infrequent relative to intra-element recombination, suggesting this process is unlikely to have a significant role in explaining the observed *A. thaliana *retrotransposable element distribution.

The ectopic exchange hypothesis makes two unique predictions for retrotransposons: solo LTRs (a product of recombination) should be observed in higher proportions relative to full-length elements outside of heterochromatin; and heterochromatic elements will show a shift toward greater average age than elements elsewhere in the genome. Our consideration of age assumes that the chance of loss by recombination remains steady or increases with element age. However, old elements will have higher sequence divergence, thereby reducing the likelihood that they will recombine. In considering age, we also assume that all elements evolve at the same rates. This is unlikely to be the case, as local, chromosomal and compartmental locations are increasingly found to have different mutation rates [36,37].

With respect to the distribution of solo LTRs, our data show exactly the opposite bias predicted by the ectopic exchange model: the ratio of Metaviridae solo LTRs to FLEs in heterochromatin was nearly twice that found outside heterochromatin. The frequency of solo LTRs at the centromeres suggests that homologous recombination, at least over short distances (less than 20 kilobases (kb)), occurs frequently in pericentromeric regions.

While we did observe the predicted shift toward older elements within heterochromatin, the data are not consistent with low rates of recombination as the determinant of retrotransposon accumulation at the centromeres. Within the Metaviridae, for example, the *Metaviruses *and *Tat *elements differ significantly in their association with heterochromatin. The ectopic exchange model would predict that the *Tat *elements should be older; however, these two lineages do not differ significantly in age. Although it is possible that recombinational forces could act differentially on different element sublineages, we view this as unlikely. Rather, forces other than ectopic recombination, such as targeted integration (see below), are responsible for the differential genomic distribution of certain element lineages. This is not to say that ectopic exchange has no role; however, it is unlikely to be the sole or prevailing influence.

### Deleterious insertion model

The deleterious insertion model hypothesizes that element insertions are generally harmful to the host, and thus elements accumulate in regions of low gene density, where insertions are least likely to have negative effects on the host. According to this model, abundance of all classes of mobile elements should inversely reflect gene density within the genome. This is supported by the observation that elements are over-represented in gene-poor pericentromeric heterochromatin and are rare over much of the chromosome arms. However, we did not observe an increase in element abundance at other gene-poor heterochromatic regions (such as the telomeres and NORs), which would be predicted by the deleterious insertion hypothesis. This model would also predict that element insertions into gene-rich regions that are tolerated by the host should act as founders or safe havens for future element insertions. This could lead to an ever-expanding area of tightly clustered and frequently nested elements in euchromatin, assuming the overall random insertion rate is greater than the rate of sequence loss through recombination. Nested clusters of elements have been reported in cereals such as maize and barley [29,30]. In *A. thaliana*, although numerous potential 'seed' insertion sites are observed along the chromosome arms, we did not detect dense clusters of nested elements at these locations.

In contrast to the deleterious insertion model, it is important to recognize that some element insertions may provide a selective advantage. Studies in *C. elegans *and rice indicate that many retrotranposons are associated with genes (63% and 20% in these species respectively) [38,39]. In *D. melanogaster*, some retrotransposon-gene associations are preserved in diverse natural populations, consistent with the hypothesis that they confer a positive selective advantage [40]. Furthermore, recent analyses in *S. pombe *suggest that the Tf1 retrotransposons may regulate expression of adjacent genes [41]. We cannot rule out a role for positive selection in the distribution of some *A. thaliana *mobile elements, but identifying such a role would require a more refined analysis of element distribution and gene associations.

### Impact of targeted integration

The observation that many LTR retroelements have non-uniform genomic distributions suggested that targeted integration may be a driver of retroelement distribution patterns [42]. Neither the deleterious insertion nor ectopic recombination models address the situation where some or all elements have evolved the ability to bias their distributions through targeted integration. The LTR retroelements of *S. cerevisiae *insert preferentially into heterochromatin or sites occupied by RNA polymerase III, and in the evolutionarily distant *S. pombe *genome, retroelements are located preferentially upstream of genes transcribed by RNA polymerase II [6-9]. Retroviruses also insert preferentially into transcribed regions, with some retroviruses favoring insertions into promoter regions [4,43].

Targeted integration could contribute significantly to the chromosomal distribution of *A. thaliana *retroelements. As in other systems, targeting may occur because elements recognize a specific chromatin state and actively insert into regions with that type of chromatin. A chromatin-targeting model has the following predictions. First, very few elements will be found outside targeted chromatin domains. For example, all heterochromatic regions such as NORs, knobs and telomeres would be occupied by the same lineage of elements if these regions share a chromatin feature recognized by that lineage. Second, different retroelement lineages may be associated with different regions of the genome if they employ different targeting strategies.

The targeting hypothesis is well supported for the Metaviridae, which on a genome-wide basis differ significantly in their chromosomal distribution from the Pseudoviridae and non-LTR retrotransposons. This is particularly true for the *Athila *and *Tat *lineages, both of which are tightly associated with pericentromeric regions. *Athila *and *Tat *elements are not found in heterochromatin regions around the telomeres, however, suggesting that telomeric and centromeric heterochromatin differ. Targeted integration to pericentromeric heterochromatin may be a general feature of the Metaviridae. Members of the Metaviridae are abundant in pericentromeric heterochromatin in many grass species [44]. Langdon *et al. *[45] suggested that an evolutionary ancient member of the Metaviridae in cereals targets to centromeric domains. Portions of a maize homolog of this element were found to co-precipitate with the centromere-specific histone CENH3, indicating an association of this element with a particular type of chromatin [46].

The Pseudoviridae and non-LTR retrotransposons differ in their genomic organization from the Metaviridae and are more loosely associated with pericentromeric regions. It may be that these element lineages do not target their integration, or they may recognize other chromosomal features, although we did not observe any association with other genome features or gene classes such as tRNA genes (data not shown). *De novo *integration events have been mapped on a chromosomal level for two tobacco Pseudoviridae elements in heterologous hosts - Tto1 in *A. thaliana *and Tnt1 in *Medicago trunculata. *In both cases these elements integrated throughout the genome, displaying some preference for genic regions [47,48]. Whether this observed distribution pattern reflects random integration or recognition of some other subtle chromosomal feature remains to be determined. Because we predict that the Metaviridae recognize pericentromeric heterochromatin, an important dataset for analysis will be maps of the various DNA methylation and histone-modification patterns for the full genome. In-depth characterization of the distribution of retroelements relative to chromatin modifications may reveal additional evidence for targeting and help to understand the impact of targeting on genome organization.

## Conclusions

Our analysis of the genomic distribution of the *A. thaliana *LTR retroelements revealed that the distribution of the Pseudoviridae and the Metaviridae is non-uniform and that they tend to cluster at the centromeres. The pericentromeric association of three Metaviridae sublineages (*Metavirus*, *Tat *and *Athila*) was significantly more pronounced than for the Pseudoviridae. Several factors are likely to contribute to the centromeric association of these elements, including target-site bias, selection against euchromatin integration and pericentromeric accumulation of elements due to suppression of recombination. For the *Tat *and *Athila *lineages, however, target-site specificity appears to be the primary factor determining chromosomal distribution. We predict that, like retroelements in yeast, the *Tat *and *Athila *elements target integration to pericentromeric regions by recognizing a specific feature of pericentromeric heterochromatin.

## Materials and methods

### RetroMap and the *A. thaliana *retroelement dataset

Reverse transcriptase amino-acid sequences (as defined by [49], see also Table 1), were used to query a database of *A. thaliana *chromosomes (TIGR version 7 January 2002) with the tblastn program (E = 1e^-10^, XML output, filtering disabled) [50]. The resulting search report was imported into RetroMap. RetroMap (to be described in detail elsewhere) provides a graphical user interface (GUI) to interactively characterize LTR retrotransposons in targeted genomes or large genomic contigs (Figure 1a). RetroMap generates a nonredundant set of database hits from BLAST results generated by a given query sequence set. Hits are merged if they directly overlap or if they align to different portions of the same query sequence. In this study, the nonredundant sequences were used to re-query the chromosome database twice more using tblastx (E = 1e^-10^, XML output, filtering disabled) to identify increasingly divergent or degenerate elements. Unique hits identified in the final round of screening were taken to represent the entire complement of retroelements in *A. thaliana*.

RetroMap assigns putative LTRs where possible for each reverse transcriptase by comparing 10 kb of DNA from each flank. This is accomplished using Blast2Sequences to identify flanking repeats [51] (Figure 1b). Direct repeats found closest to the reverse transcriptase, larger than 50 bp and less than 5 kb, are considered to be LTRs. Hits with putative LTRs were considered to be full-length elements (FLE) or complete elements. Twenty-six reverse transcriptase hits were excluded from the FLEs owing to difficulty in automatic LTR assignment (13 each from the Pseudoviridae and Metaviridae). Among these were nested elements and tandem elements sharing a LTR. Reverse transcriptases were assigned to a retroelement lineage (Metaviridae, Pseudoviridae or non-LTR retrotransposon) on the basis of their similarity to the diagnostic reverse transcriptase query sequences. Full-length Metaviridae elements were further subdivided into the classic (*Metavirus*), *Tat *and *Athila *groups on the basis of the highest-scoring match in a BLAST database containing the Metaviridae reverse transcriptase sequences described in [18]. Putative complete elements with a predicted reverse transcriptase failing to significantly match any sequence in this database were removed from further consideration as false positives (two cases).

Solo LTRs and solo LTR fragments were identified with blastn (E < 1e^-5^) using all predicted 5' LTRs of known *A. thaliana *elements and the FLEs. RetroMap assigns any putative LTR sequence that fails to match or overlap with a predicted FLE LTR as a solo LTR.

### Relative age calculation for full-length elements

LTRs are identical at the time of retroelement integration, and so relative element ages were estimated from the percentage of identical residues shared between 5' and 3' LTRs for FLEs. The age formula used was *T *= *d*/2*k *(time (*T*) = genetic distance (*d*)/ [2 × substitution rate (*k*)]), where genetic distance is 1 - (percent identity/100) and the substitution rate is 1.5 × 10^-8 ^[52].

### Assignment of heterochromatin boundaries

Chromosome coordinates relative to the left (north) end were used to calculate distances between retroelements and heterochromatic domains. Heterochromatin boundaries were derived from [20-22] and include the telomeres, heterochromatic knobs, NORs and centromeres. Chromosome end-coordinates were considered as the telomere boundaries. The *A. thaliana *NORs are located at the left (north) ends of chromosomes 2 and 4, and as these regions were only sample sequenced, their boundaries were assigned as the left ends of chromosomes 2 and 4. Heterochromatic knobs and pericentromeric regions were assigned as the outermost physical markers delimiting these regions, as determined by the studies listed above.

### Statistical tests

A RetroMap-generated datafile was used as the data source for statistical testing. The data file contains chromosomal element coordinates, LTR identity, age and lineage information for all *A. thaliana *retroelement families by element category: reverse transcriptase only (R), full-length (F), and solo LTR (S).

For each element type and each chromosome, a Kendall-Sherman test [53-55] was conducted to determine if the element positions were randomly distributed across chromosomes according to a uniform distribution. A permutation test [55] was used to assess the statistical significance of observed differences in the chromosomal position distributions for each chromosome across various element categories. The multi-response permutation procedures (MRPP) test is briefly described as follows. The average distance between a pair of elements within a category of interest is determined. A weighted sum of these averages over all categories of interest is computed, with each category weighted in proportion to the number of elements in the category. This weighted sum is the observed value of the test statistic. Next, the test statistic is re-computed for each of 10,000 random permutations of the category labels. For each permutation, the observed chromosomal positions of the elements are held constant while the category labels are randomly shuffled. The proportion of the 10,000 permutation-replicated test statistics that are less than or equal to the original observed test statistic serves as an approximate *p*-value for a test whose null hypothesis is that all element categories of interest have the same chromosomal position distribution. This permutation approach is useful for the chromosomal position data because first, no distributional assumptions are required, second, differences in chromosomal position distributions other than simple location shifts are detectable, and third, the method is not as sensitive to outliers as common parametric approaches.

For FLEs, linear model analyses were used to assess the effects of the factors 'chromosome', 'lineage/sublineage', and 'location' relative to heterochromatin on the response variable 'element age'. *F*-tests were used to check for interaction between these three factors and to assess the statistical significance of observed differences among the five chromosomes, among the four lineage/sublineage categories (Pseudoviridae and the three Metaviridae sublineages:*Athila*, *Tat *or *Metavirus*), and between elements inside and outside heterochromatin. The square root of age was used as the response variable in the age analysis so that the variance of the response would be roughly constant across categories defined by combinations of chromosome, lineage/sublineage, and location, as required for standard linear model analyses. Outlying observations were present, but the results of the analysis remained essentially the same with or without the outliers. Thus the reported results are based on the full dataset.

## Additional data files

The following additional data are available with the online version of this article: a Microsoft Excel spreadsheet of data generated by RetroMap for each retrotransposon insertion identified; the data in this file was used for all statistical analyses (Additional data file 1). The Java application used to generate the LTR and retrotransposon coordinates and to estimate retrotransposon ages (Additional data file 2). To run RetroMap, version 1.3 or higher of the Java Runtime Environment (JRE ) must be present. To enable searches for LTRs, NCBI's BLAST 2 Sequences must be locally installed.

## Supplementary Material

Additional data file 1 A Microsoft Excel spreadsheet of data generated by RetroMap for each retrotransposon insertion identified; the data in this file was used for all statistical analyses Click here for additional data file

Additional data file 2 The Java application used to generate the LTR and retrotransposon coordinates and to estimate retrotransposon ages Click here for additional data file

## Figures and Tables

**Table 1:** Retroelement species used as BLAST probes

Element	GenBank accession number	Host organism	Family	Genus	Length (nucleotides)	LTR identity (length in nucleotides)
*Athila4*-6	AF296831	*Arabidopsis thaliana*	MV	*Metavirus*	14,016	98.2 (1747)
Cer1	U15406	*Caenorhabditis elegans*	MV	*Metavirus*	8,865	100.0 (492)
*Osvaldo*	AJ133521	*Drosophila buzzatii*	MV	*Metavirus*	9,045	99.9 (1196)
*Sushi*	AF030881	*Fugu rubripes*	MV	*Metavirus*	5,645	91.0 (610)
Tf1	M38526	*Schizosaccharomyces pombe*	MV	*Metavirus*	4,941	100.0 (358)
Ty3	M23367	*Saccharomyces cerevisiae*	MV	*Metavirus*	5,428	100.0 (340)
Art1	Y08010	*A. thaliana*	PV	*Pseudovirus*	4,793	99.8 (439)
*Copia*	M11240	*Drosophila melanogaster*	PV	*Hemivirus*	5,416	100.0 (276)
*Endovir1*-1	AY016208	*A. thaliana*	PV	*Sirevirus*	9,089	99.8 (548)
*SIRE*-1	AF053008	*Glycine max*	PV	*Sirevirus*	10,444	100.0 (2149)
Tca2	AF050215	*Candida albicans*	PV	*Hemivirus*	6,428	100.0 (280)
Tca5	AF065434	*C. albicans*	PV	*Hemivirus*	5,588	100.0 (685)
*Jockey*	M22874	*D. melanogaster*	NL	-	5154	-
L1.2	M80343	*Homo sapiens*	NL	-	6,050	-
R1	X51968	*D. melanogaster*	NL	-	5356	-
R2	X51967	*D. melanogaster*	NL	-	3,607	-
Ta11	L47193	*A. thaliana*	NL	-	7,808	-

MV, Metaviridae; PV, Pseudoviridae; NL, non-LTR retrotransposon.

**Table 2:** *A. thaliana *LTR retroelements by chromosome

	Chromosome 1 30,080,809 nucleotides	Chromosome 2 19,643,621 nucleotides	Chromosome 3 23,465,812 nucleotides	Chromosome 4 17,549,528 nucleotides	Chromosome 5 26,689,408 nucleotides	Total 117,429,178 nucleotides
**Pseudoviridae**						
RT only	21	19	16	10	16	82
Complete elements	48	42	47	35	38	210
Nucleotides	239,675	211,083	285,207	185,127	199,386	1,120,478
Percentage of total nucleotides	0.88%	1.24%	1.34%	1.21%	0.96%	1.1%
Solo LTRs	84	100	125	89	87	485
Nucleotides	16,516	19,275	23,906	15,500	15,248	90,445
Percentage of total nucleotides	0.13%	0.16%	0.18%	0.15%	0.13%	0.15%
						
**Metaviridae**						
RT only	16	30	41	23	32	142
Complete elements	37	34	45	18	32	166
Nucleotides	309,690	319,802	375,703	161,352	319,535	1,486,082
Percentage of total nucleotides	1.23%	2.82%	2.22%	1.40%	1.59%	1.74%
Solo LTRs	435	500	928	360	560	2,783
Nucleotides	228,115	257,810	326,484	179,500	262,187	1,254,096
Percentage of total nucleotides	1.15%	1.74%	1.71%	1.42%	1.24%	1.42%
						
***Athila***						
Complete elements	7	8	8	4	11	38
Nucleotides	72,094	90,171	93,015	37,339	119,646	412,265
Percentage of total nucleotides	0.38%	0.87%	0.67%	0.41%	0.69%	0.60%
						
***Tat***						
Complete elements	14	10	8	6	8	46
Nucleotides	131,154	102,534	83,327	68,754	103,112	591,944
Percentage of total nucleotides	0.44%	0.54%	0.52%	0.46%	0.56%	0.50%
						
***Metavirus***						
Complete elements	16	16	29	8	13	82
Nucleotides	106,442	127,097	199,361	55,259	96,777	748,231
Percentage of total nucleotides	0.42%	1.03%	1.03%	0.52%	0.33%	0.64%
						
**Non-LTR retrotransposon**	49	90	69	32	71	311
						
**Total LTR contribution**						
Complete elements	85	76	92	53	70	376
Nucleotides	634,695	798,606	836,968	457,405	679,255	3,331,357
Percentage of total nucleotides	2.11%	4.07%	3.57%	2.61%	2.55%	2.84%
Solo LTRs	519	600	1,053	449	647	3,268
Nucleotides	386,759	373,256	444,804	275,361	364,340	1,844,520
Percentage of total nucleotides	1.29%	1.90%	1.90%	1.57%	1.37%	1.57%
Both						
Nucleotides	1,021,454	1,171,862	1,281,772	732,766	1,043,595	5,175,877
Percentage of total nucleotides	3.40%	5.97%	5.46%	4.18%	3.91%	4.41%

**Table 3:** Comparison of genome localization by retroelement lineage

Hypotheses	Test	Group(s) tested	*p*-values by chromosome					Accept?
				
			1	2	3	4	5	
All families are randomly distributed according to a uniform distribution	Uniform goodness of fit, 10,000 random permutations	MV(F)	**0.0000**	**0.0000**	**0.0000**	**0.0000**	**0.0000**	No
		PV(F)	**0.0000**	**0.0007**	**0.0000**	**0.0022**	**0.0464**	No
		MV(S)	**0.0000**	**0.0000**	**0.0000**	**0.0000**	**0.0000**	No
		PV(S)	**0.0000**	**0.0000**	**0.0000**	**0.0000**	**0.0000**	No
		MV(R)	**0.0000**	**0.0000**	**0.0000**	**0.0000**	**0.0000**	No
		PV(R)	**0.0000**	**0.0007**	**0.0000**	**0.0097**	**0.0000**	No
		NL(R)	**0.0000**	**0.0000**	**0.0000**	**0.0002**	**0.0000**	No
								
Retroelement family distributions are organized similarly in the genome	MRPP, 10,000 random permutations	MV(FSR), PV(FSR), NL(R)	**0.0000**	**0.0000**	**0.0000**	**0.0000**	**0.0000**	No
		MV(FSR), PV(FSR)	**0.0000**	**0.0000**	**0.0000**	**0.0000**	**0.0000**	No
		MV(FSR), NL(R)	**0.0000**	**0.0000**	**0.0000**	**0.0000**	**0.0000**	No
		PV(FSR), NL(R)	0.3498	0.8326	**0.0241**	0.1468	0.1417	Yes
								
All Metaviridae sublineages have similar distributions	MRPP, 10,000 random permutations	MV *Athila*, *Metavirus*, *Tat*	0.2200	0.1365	0.5676	0.4174	0.2788	Yes
		MV *Athila*, *Metavirus*	0.1057	0.3010	0.2657	0.4526	0.4453	Yes
		MV *Athila*, *Tat*	0.1687	0.0970	0.7116	0.3773	0.2781	Yes
		MV *Metavirus*, *Tat*	0.4903	0.1268	0.7341	0.5753	0.2361	Yes
								
Metaviridae subtypes have similar distributions	MRPP, 10,000 random permutations	MV(FSR)	0.7742	0.1247	**0.0000**	0.7425	0.0659	Yes
		MV(FS)	0.4544	0.1357	**0.0003**	0.4435	0.7241	Yes
		MV(FR)	0.5184	0.9461	0.5750	0.5480	0.2135	Yes
		MV(SR)	0.9068	0.1339	**0.0051**	0.8194	**0.0157**	Yes
								
Pseudoviridae subtypes have similar distributions	MRPP, 10,000 random permutations	PV(FSR)	0.0509	0.2039	0.2199	0.0953	**0.0379**	Yes
		PV(FS)	0.2732	0.0853	0.2665	0.6567	**0.0453**	Yes
		PV(FR)	**0.0136**	0.5055	0.1185	0.0521	**0.0281**	Yes
		PV(SR)	0.0743	0.5604	0.2513	**0.0307**	0.3476	Yes

MV, Metaviridae; PV, Pseudoviridae; NL, non-LTR retrotransposon; R, RT-only; S, solo LTR; F, full-length element. *p*-values < 0.05 are displayed in bold text.

**Table 4:** Association of retroelements with heterochromatin

Hypotheses	Test	Group(s) tested	*p*-values	Accept?
All families share a similar probability of being in or outside heterochromatin	MRPP, 10,000 random permutations	MV(FSR), PV(FSR), NL(R)	**0.0000**	No
		MV(FSR), PV(FSR)	**0.0000**	No
		MV(FSR), NL(R)	**0.0000**	No
		PV(FSR), NL(R)	**0.0000**	No
				
Metaviridae sublineages have similar heterochromatic distributions	MRPP, 10,000 random permutations	MV *Athila*, *Metavirus*, *Tat*	**0.0011**	No
		MV *Athila*, *Metavirus*	**0.0016**	No
		MV *Athila*, *Tat*	0.5211	Yes
		MV *Metavirus*, *Tat*	**0.0105**	No
				
Element subtypes have similar heterochromatic distributions	MRPP, 1,000 random permutations	MV(SR), PV(SR), NL(R)	**0.0000**	No
		MV(FR), PV(FR), NL(R)	0.3960	Yes
		MV(FS), PV(FS)	**0.0000**	No
				
Pseudoviridae subtypes have similar heterochromatic distributions	Pearson's chi-square	PV(FSR)	**0.0002**	No
		PV(FS)	**0.0001**	No
		PV(FR)	**0.0073**	No
		PV(SR)	0.9419	Yes
				
Metaviridae subtypes have similar heterochromatic distributions	Pearson's chi-square	MV(FSR)	**0.0001**	No
		MV(FS)	**0.0002**	No
		MV(FR)	0.5146	Yes
		MV(SR)	**0.0159**	No

MV, Metaviridae; PV, Pseudoviridae; NL, non-LTR retrotransposon; R, RT-only; S, solo LTR; F, full-length element. *p*-values < 0.05 are displayed in bold text.

**Table 5:** Comparison of LTR retroelement age distributions

Hypotheses	Test	Group(s) tested	Chromosome *p*-values	Accept?
Metaviridae and Pseudoviridae have similar age distributions in the genome	MRPP, 10,000 random permutations	MV(F), PV(F)	**0.0000**	No
				
Metaviridae sublineages have similar age distributions	MRPP, 10,000 random permutations	MV *Athila*, *Metavirus*, *Tat*	**0.0000**	No
		MV *Athila*, *Metavirus*	**0.0000**	No
		MV *Athila*, *Tat*	**0.0003**	No
		MV *Metavirus*, *Tat*	0.4618	Yes
				
Metaviridae age distributions are similar whether the elements are in or out of heterochromatin	MRPP, 10,000 random permutations	MV(F)	**0.0021**	No
				
Metaviridae sublineage age distributions are similar whether they are in or outside heterochromatin	MRPP, 10,000 random permutations	MV *Athila*	**0.0410**	No
		MV *Metavirus*	0.5747	Yes
		MV *Tat*	**0.0457**	No
				
Pseudoviridae age distributions are similar whether the elements are in or outside heterochromatin	MRPP, 10,000 random permutations	PV(F)	**0.0167**	No

MV, Metaviridae; PV, Pseudoviridae; NL, non-LTR retrotransposon; R, RT-only; S, solo LTR; F, full-length element. *p*-values < 0.05 are displayed in bold text.
